# Workforce Agility: Development and Validation of a Multidimensional Measure

**DOI:** 10.3389/fpsyg.2022.841862

**Published:** 2022-03-25

**Authors:** Moritz K. H. Petermann, Hannes Zacher

**Affiliations:** ^1^Wilhelm Wundt Institute of Psychology, Universität Leipzig, Leipzig, Germany; ^2^Daimler AG, Stuttgart, Germany

**Keywords:** workforce agility, performance, well-being, job satisfaction, agile project management

## Abstract

The concept of workforce agility has become increasingly popular in recent years as agile individuals are expected to be better able to handle change and uncertainty. However, agility has rarely been studied in a systematic way. Relations between agility and positive work outcomes, such as higher performance or increased well-being, have often been suggested but rarely been empirically tested. Furthermore, several different workforce agility measures are used in the literature which complicates the comparison of findings. Recognizing these gaps in the literature, we developed a new workforce agility measure, compared this measure to established workforce agility measures, and empirically tested the relations of workforce agility with work outcomes. For this purpose, we surveyed participants from two samples (*N*_1_ = 218, *N*_2_ = 533). In a first step, we used Sample 1 to examine the factor structure of the measure for item selection. In a second step, we used Sample 2 to confirm the 10-factor structure and to compare the predictive validity of our measure along with two other agility measures. Findings demonstrate predictive validity for all three workforce agility scales, especially in relation to innovative performance. Furthermore, workforce agility related positively to task and innovative performance, organizational citizenship behavior, job satisfaction, and well-being.

## Introduction

Increasingly, unpredictable and far reaching change is shifting the context organizations are currently operating in (Dyer and Shafer, 2003). Due to a soaring level of globalization, higher customer expectations, and an elevated tempo of innovation, markets are getting increasingly dynamic, competitive, and challenging (Breu et al., 2002). In an attempt to overcome these challenges, an increasing number of organizations tend to rely on agile workforces (Sherehiy and Karwowski, 2014), as these have been suggested to provide several benefits to the organization. Agile workforces have been described to be more responsive and competent, as well as to be better able to adapt to new surroundings and circumstances (Zhang and Sharifi, 2000; Breu et al., 2002). They are also expected to boost individual performance (Laanti, 2013; Braun et al., 2017), business growth in the events of unanticipated and constant change (Gehani, 1995), and increase productivity (Goldman et al., 1995). Along with this, it has been assumed that workforce agility has a positive impact on the individuals working in the organization. Employees are expected to have higher levels of well-being, less stress during their work (Laanti, 2013), and a greater overall job satisfaction (Melnik and Maurer, 2006). Consequently, it might be of great value to an organization to build up and foster an agile workforce.

Despite the impact workforce agility is expected to have on organizational functioning as well as the individual employee, it has rarely been studied empirically (Sherehiy and Karwowski, 2014; Muduli and Pandya, 2018). Workforce agility is most often described to consist of two factors: (a) the ability to properly respond to change and (b) the ability to exploit this change (Chonko and Jones, 2005; Alavi et al., 2014). Several workforce agility models and measures are currently used in the agility literature; however, they have often been criticized for their conceptual vagueness. Particularly, Sherehiy (2008) often used three-dimensional framework has been the target of critics as it did not show a good model fit in the original study. Other models such as the model of Petermann and Zacher (2021) lack a measurement and have not been empirically tested. Petermann and Zacher (2021) describe workforce agility as consisting of 10 different dimensions: (a) accept changes, (b) decision making, (c) create transparency, (d) collaboration, (e) reflection, (f) user centricity, (g) iteration, (h) testing, (i) self-organization, and (j) learning. Furthermore, the anticipated positive outcomes such as higher well-being, performance, or satisfaction have only rarely been the target of empirical research. Research so far largely focused on factors influencing agility (Alavi et al., 2014; Sherehiy and Karwowski, 2014; Muduli and Pandya, 2018) and the outcomes of agile methodologies such as scrum (Li et al., 2010; Moe et al., 2010; Laanti, 2013; Tripp et al., 2016; Tuomivaara et al., 2017). It is, therefore, fundamental to compare the different agility measures and to examine the outcomes of an agile workforce on the basis of an empirical study.

**Figure 1:**
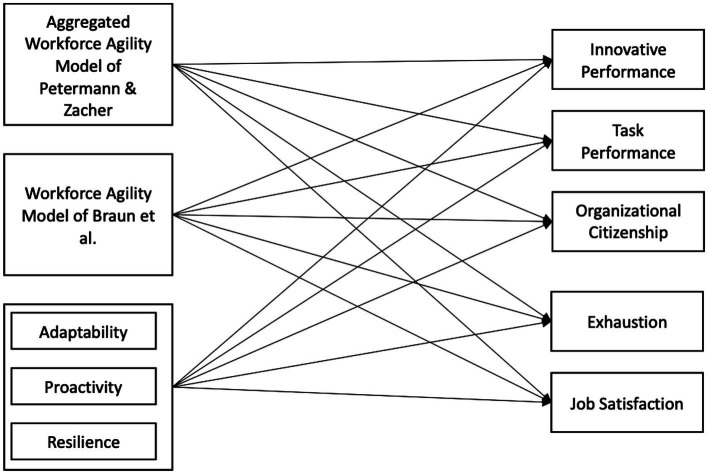
Research model for sample 2.

Recognizing the current discussion about workforce agility measures, we first aim to develop a new measure for workforce agility based on the model of Petermann and Zacher (2021). We will then compare the newly created measure to the two established workforce agility measures of Braun et al. (2017) and Cai et al. (2018) in regard to predictive validity and model fit. In a third step, we will examine the hypothesis that workforce agility has positive relationship with positive work-related outcomes. Especially the relation of workforce agility to performance (Plonka, 1997; Sherehiy and Karwowski, 2014; Braun et al., 2017) and well-being (Mannaro et al., 2004) was often suggested by the agility literature. Based on this, as well as self-determination theory (Ryan and Deci, 2000), the job characteristics model (Oldham et al., 1976), and the job demand-control model of Karasek (1979), we hypothesize that workforce agility has a positive relation to task performance, innovation performance, organizational citizenship behavior, employee well-being, and job satisfaction. We will test these hypotheses using structural equation modeling with the three workforce agility measures as independent variables and work-related outcomes as dependent variables (Figure 1).

Considering that agility and especially the development of an agile workforce is supposed to have a multitude of benefits for the organization as well as the individual employee, it is important that an increased amount of research focuses on this topic. We argue that research and practice will benefit from this study as it offers a new and more specified measurement of workforce agility and compares three workforce agility measures based on their predictive validity toward work-related outcomes. Combined with a clear discussion of the advantages and disadvantage of each scale, this might support future research and organizational practice in choosing the appropriate scale to measure agility in their context. Additionally, this research will increase the understanding of the outcomes of workforce agility in that it will give empirical insights into the proposed relationships of agility with employee well-being, job satisfaction, and performance factors such as task and innovative performance as well as organizational citizenship behavior. These relationships have often been suggested by research, however, have not yet been examined by quantitative research. A clear understanding of the outcomes of agility that can be attained by empirical research might be especially important since many organizations are currently changing their business model to incorporate a more agile way of working to profit from the suggested benefits. Consequently, the agility research is critical to the practitioners in organizations and should focus on explaining the underlying behaviors and structures as well as the outcomes of workforce agility. Our research is, hence, of great value for agility research as well as organizations which seek to understand these relationships better, as it offers them a first orientation about what to expect from a shift toward a more agile workforce.

### Workforce Agility

Workforce agility is described by a multitude of different definitions, theories, and conceptualizations in the current literature. It has been described as an ability, as an attitude, and as a set of specific behaviors (Muduli and Pandya, 2018; Salmen and Festing, 2021). However, there seems to be some agreement to describe it as consisting of two main aspects: (a) the ability of a workforce to properly respond to change and (b) the ability to exploit changes to use them as opportunities (Chonko and Jones, 2005; Alavi et al., 2014). This section will provide a short overview over the different conceptualizations currently used in the agility literature.

Breu et al. (2002) described one of the first empirically tested models of workforce agility consisting of the five agile capabilities intelligence (interpret change and being responsive to customer and market needs), competencies (quickly developing new skills and gaining new information), collaboration (effectively cooperating across functional boundaries and projects), culture (empowerment of agility to make independent decisions), and information systems (support of IT infrastructure).

Other models described workforce agility from a behavioral perspective (Sherehiy and Karwowski, 2014) and defined agility as observable performance or behavior at work consisting of the three different dimensions proactivity, adaptability, and resilience. Proactivity consists of initiating behaviors (initiating activities that lead to a solution of a change-related problem) and anticipation behaviors (sensing and anticipating problems) and describes an individual’s activities that have a positive impact on the environment. Adaptability consists of learning behaviors (constantly learning new tasks, skills, and procedures), interpersonal adaptability (being able to get along and work with individuals from different professions and backgrounds), and professional flexibility (assuming and changing different roles when needed) and describes an individual’s modification to oneself to become a better fit to the environment (Griffin and Hesketh, 2003; Sherehiy and Karwowski, 2014). Lastly, resilience describes an individual’s ability to cope in a changing environment and to function effectively in stressful situations. This model is currently very popular in the workforce agility literature and was widely adopted in different research projects (Alavi et al., 2014; Cai et al., 2018; Muduli and Pandya, 2018). Researchers like Alavi et al. (2014) showed that organizational learning, flat organizational structures, decentralization of decision making, as well as psychological availability, meaningfulness, and safety positively relate to workforce agility (Cai et al., 2018). Furthermore, they show that relationship conflict has a negative and task conflict a positive influence on workforce agility (Liu et al., 2015a).

However, there were some conceptual problems with the three-dimensional model which led to several other models of agility currently used in the literature. Muduli and Pandya (2018), for example, used an adapted model of Breu et al. (2002) and considered an agile workforce to be flexible, adaptive, proactive, and resilient but also, to have a positive attitude toward learning and change, to be good problem solvers, very innovative, and to be able adapt new responsibilities, and generate novel ideas. Following the approach of Muduli and Pandya (2018) to define workforce agility as consisting of proactive and adaptive dimensions, Braun et al. (2017) defined workforce agility as the skill of individuals to proactively overcome obstacles or create opportunities by rethinking usual approaches. They argued that agile individuals constantly monitor the environment to be able to anticipate and quickly respond to change. Based on this definition, they developed a five-item measurement of workforce agility. Similarly, recent research has provided evidence that workforce agility is a multidimensional construct that can be assessed *via* different dimensions of agility. They considered adaptability and proactivity as agile dimension but subcategorized these dimensions further into the subdimensions resilience, teamwork, coping with change, decisiveness, eagerness to learn, independence, and courage (Doeze Jager-van Vliet et al., 2019). Furthermore, Harsch and Festing (2020) defined agile talents as innovative, mobile, customer orientated, resilient, and adaptable people with a high willingness to learn and perform and who constantly question the status quo.

Recognizing this multitude of different definitions in the agility literature, Petermann and Zacher (2021) developed a taxonomy of the agile workforce. This taxonomy was developed based on concept mapping as well as the critical incident technique and consists of 10 different dimensions. These dimensions include as: (a) accept changes, (b) decision making, (c) create transparency, (d) collaboration, (e) reflection, (f) user centricity, (g) iteration, (h) testing, (i) self-organization, and (j) learning. For the exact definitions of the 10 dimensions, please see Table 1. All of these dimensions relate to a formative higher order factor workforce agility which will be the base of the conducted research. In contrast to reflective measures, the direction of the relationship in formative measures runs from the measure to the construct and not vice versa (Diamantopoulos et al., 2008). We used a formative higher order factor instead of a reflective factor as we suspect that the 10 dimensions are causes of workforce agility rather than caused by it. This means that we argue that workforce agility, or in other words the ability to correctly respond to and exploit change, is caused by behaviors such as quick decision making, cooperation, or reflection instead of the other way around.

**Table 1:** The workforce agility model of Petermann and Zacher (2021).

Dimensions	Description
Accept changes	This dimension concerns the revision of previous decisions due to other new information as well as the acceptance of different roles and situations. It further contains the ability to flexible, quickly and successfully adapt to changing circumstances.
Decision making	This dimension concerns the ability of people to tolerate risks, prioritize, react and decide quickly and proactively. It further contains the ability of people to take responsibility for their actions.
Create transparency	This dimension concerns quickly sharing information, admitting to mistakes, asking for help or information as well as direct communication preferably face to face.
Collaboration	This dimension concerns the creation of agreements as well as the adherence to these agreements and to rules. It further contains that remembers go along with decisions that were made by the team and trust each other. It suggests a collaboration that is cross functional, open, dynamic and works beyond team boarders. Lastly it concerns the deferment from egoistic behavior, the valuing of others and an empathic behavior.
Reflection	This dimension concerns questioning current behaviors, reflecting the collaboration and constantly looking for improvements in the work.
User centricity	This dimension concerns constantly integrating the customer in the project and collecting and including feedback of the customer. It further, puts the value for the customer in the center of attention and integrates them in the development process.
Iteration	This dimension concerns developing a project in a stepwise manner, make continuous improvements and to act in short adaptive cycles.
Testing	This dimension concerns the regular testing of a product as well as the building of a prototype, experimenting and trying out new things. It does not include the test of a method.
Self-organization	This dimension concerns the commitment of the team members and the willingness to manage structure and organize themselves.
Learning	This dimension contains the necessity for constant education as well as a good knowledge management and the possibility to learn from others.

### Workforce Agility and Performance

Research has provided evidence that work performance consists of more than the direct fulfillment of one’s job but also of, for example, driving change in the organization or cooperating with others (Welbourne et al., 1998). It can be investigated as a single higher order factor or as a multidimensional construct (Viswesvaran and Ones, 2000). In this research, we will use a multidimensional construct instead of one global factor as we want to examine the influence of workforce agility on the different performance dimensions. We expect workforce agility to be positively related to specific work performance factors such as innovative performance, task performance, and organizational citizenship behavior. For this, we will consider three different psychological theories: First, we will consider self-determination theory (Ryan and Deci, 2000), second, we will consider the job characteristics model of Oldham et al. (1976), and third, we will consider the job demand-control model of Karasek (1979).

Self-determination theory argues that humans possess three basic psychological needs: the need for autonomy, the need for competence, and the need for relatedness (Ryan and Deci, 2000). These needs have been shown to be positively related to several positive work outcomes such as performance (Gagne and Deci, 2005; Deci and Ryan, 2008). We argue that workforce agility is directly related to the psychological needs described by the self-determination theory and with that relate to employee performance. An example of this relation would be the agile behavioral dimensions of learning, reflection, or decision making that might be positively related to the need for competence.

Additionally, we argue that workforce agility relates to different dimensions of the job characteristics model of Oldham et al. (1976). The job characteristics model describes five core job dimensions: skill variety, task identity, task significance, autonomy, and feedback. These have also been associated with positive job outcomes such as performance, work satisfaction, or well-being (Oldham et al., 1976; Fried and Ferris, 1987). We argue that workforce agility enriches job tasks of employees, as agile work behaviors provide constant feedback from customers as well as team members through reflection practices and user inclusion. Furthermore, it grants a great amount of autonomy through self-organizing mechanisms and behaviors, highlights the significance of the individual tasks by constantly relating them back to the whole project, requires a great variability of tasks such as customer communication, project management, or product testing, and creates identifiable work pieces through short and stepwise work cycles. Subsequently, we propose that workforce agility relates to positive work outcomes such as performance, satisfaction, or well-being.

Finally, we will consider the job demand-control model of Karasek (1979). This model argues that psychological strain results from the joint effect of the job demand and the control, e.g., the decision-making freedom the worker has over the situation. The individuals control over the situation is the variable that distinguishes if a worker experiences high stress or if the worker experiences the work as an active job, when faced with high job demands. High control in the form of a high decision-making latitude has been related to a lower level of work stress and consequently a higher well-being of workers, but also to several other positive work outcomes such as performance, organizational commitment, or task enjoyment (Karasek, 1979; Bakker et al., 2010). We argue that workforce agility positively relates to the degree of control felt by the worker as different dimensions such as decentralized decision making or self-organization are continuously named as dimensions of agility. Similarly to our argumentation based on self-determination theory and the job characteristics model, the consideration of the job demand-control model leads us to the expectation that workforce agility has a positive relation to different work outcomes such as performance or well-being.

In line with the theoretical background, research has often proposed that workforce agility is related to different performance criteria. For instance, Liu et al. (2015b) who found a positive relation of team agility on task performance of teams. This is also supported by the finding of Braun et al. (2017), who found that individual agility significantly influences and predicts the supervisor rated performance of employees. In addition to task performance, workforce agility has often been related to innovative performance (Doeze Jager-van Vliet et al., 2019; Harsch and Festing, 2020). Individuals in an agile workforce have been described to be good problem solvers, to be able to quickly learn new skills, to be highly innovative, and to be better able to cope with new situations, setbacks, or uncertainty (Plonka, 1997; Sherehiy and Karwowski, 2014; Braun et al., 2017). In line with this description of an agile workforce, Abrishamkar et al. (2021) found that workforce agility positively relates to product innovation and consequently to a higher likelihood of an organization becoming a high-tech manufacturing firm. On an organizational level, McCann et al. (2009) found a significant positive relationship between organizational agility and performance. They argued that agility makes companies more competitive which in turn leads to an increase of profitability. Similarly, Pulakos et al. (2019) found the organizational characteristics stability, rightsized teamwork, and relentless course correction to be directly related to agility which in turn had a positive effect on the financial performance of a company. Lastly, research considering agile methodologies showed a relation between agility and performance. Laanti (2013) found that 64% of teams that were adapting to the agile methodologies scrum or Kanban felt that their performance did increase after taking the methodologies into use. Peeters et al. (2022) studied 97 agile teams and found that the agile way of working positively relates to team performance and engagement. Similarly, a case study by Mann and Maurer (2005) observed a higher subjective work quality as well as an increased customer satisfaction for teams working with the agile methodology scrum, over the course of 2 years. These results were also supported by a case study by Li et al. (2010), who found that the introduction of scum in an organizational setting led to a more efficient software development process as well as higher quality. Contrary to these findings, Annosi et al. (2015) found that time pressures that were induced by the implementation of agile methodologies, especially scrum methodology, can lead to less team engagement in learning and innovation activities.

Taken together, we expect agility to be positively related to the performance factors task performance, organizational citizenship behavior, and innovative performance. Task performance concerns the quality, quantity, and accuracy of work as well as the customer service. We expect workforce agility to positively relate to task performance as agile individuals have been seen to produce higher quality of work (Mann and Maurer, 2005) and have been found to be better able to observe, anticipate, and meet customer needs (Chonko and Jones, 2005). Organizational citizenship behavior concerns helping others and working for the overall good of the company. We argue that agility relates to organizational citizenship behavior as it was proposed that an agile workforce is better able to collaborate across functional, team, and department boarders to utilize all existing recourses (Sherehiy and Karwowski, 2014). Lastly, innovative performance concerns finding and implementing new ideas or new ways of working. We argue that workforce agility has a strong positive relationship with innovative performance as an agile workforce regularly reflects their processes and searches for improvements, continuously tests and experiments with new ideas, and acts in short iterations (Petermann and Zacher, 2021).

*H1*: Workforce agility is positively related to (a) task performance, (b) organizational citizenship behavior, and (c) innovative performance.

### Workforce Agility and Well-Being

Following our argumentation about workforce agility and its relation to self-determination theory, the job characteristics model, and the job demand-control model, we expect agility to be positively related to well-being and job satisfaction. All of these models have been linked to several positive work outcomes including an increased job satisfaction, lower emotional distress, and a lower likelihood to burn out (Karasek, 1979; Spector, 1986; Fried and Ferris, 1987; Schaufeli and Bakker, 2004; Deci and Ryan, 2008). This is also supported by the findings of Tripp et al. (2016), who found that agile methodology positively influences the job design criteria job autonomy, feedback, skill variety, task identity, and task significance. We, therefore, expect a positive relationship between workforce agility and well-being.

This is partially supported by the current literature. Whereas Braun et al. (2017) found an interaction effect of resilience and agility on stress and proposed that an increase of agility without a simultaneous increase of resilience might have a negative impact on employees stress level, other authors did not find any effect of agility on stress levels (Laanti, 2013) or observed a positive relationship between the use of agile methodology and well-being (Mannaro et al., 2004; Tuomivaara et al., 2017). Mannaro et al. (2004) found that the adoption of an agile method led to higher job satisfaction and to lower perceived stress of the employees. Furthermore, Tuomivaara et al. (2017) argued that agile teams were able to work in a more sustainable pace which led to better recovery times and to lower perceptions of stress in the end of working periods. In line with this, Mann and Maurer (2005) found that the amount of overtime decreased in projects that adopted an agile method as the new way of working and that the working mode shifted toward a more sustainable pace. Lastly, Rietze and Zacher (2022) found that agile work practices have a negative effect on emotional fatigue by lowering work demands and a positive effect on emotional engagement through higher job resources. As the current state of the literature is somewhat inconclusive, we will examine the relationship between workforce agility and well-being further in this research. Well-being at work has been indexed by a number of variables. For this study, we choose job satisfaction and job exhaustion.

*H2*: Workforce agility is positively related to (a) job satisfaction and negatively related to (b) job exhaustion.

## Materials and Methods

### Participants and Procedure

We used two different samples for this research. The first sample was used for a first confirmatory factor analysis to test of our proposed workforce agility construct and adapt the measure on the basis of this test. The second sample was used to validate these adaptions and to examine the relations of workforce agility with the outcome performance, well-being, and job satisfaction.

Sample 1 consisted of 218 participants of which 77 were managers and 141 were employees that worked in the production of a big manufacturing company. The participants were invited to participate *via* an email link by the head of human resources, using the third-party survey provider “Interview.” Participants were asked to fill the online survey on their work computers during their workday and answer a number of questions about themselves and their work. Please see Table 2 for the demographic data. Due to data protection, reasons of the company questions about organizational tenure and gender were not asked in this survey.

**Table 2:** Demographic data.

	Sample 1 (%)	Sample 2 (%)
**Age in years**		
Under 25	2	3
25–34	17	20
35–44	20	22
45–54	31	30
55–60	22	20
**Degree**		
Doctoral	6	7
Master’s	24	40
Bachelor’s	11	21
High school	4	9
Middle school	17	17
General education	4	6
**Gender**		
Female	-	41
Male	-	59

Sample 2 consisted of 533 participants of which 160 were managers and 373 were employees that either worked in a big manufacturing company and were invited to participate *via* an email link by the head of human resources (412 participants) or were recruited *via* a free link in an online network for systemic coaches (121 participants). All participants were asked to fill in the online survey using the third-party survey provider “Interview” and answer a number of questions about themselves and their work. Please see Table 2 for the demographic data. Due to data protection reasons of the manufacturing company, only the participants recruited from the systemic network were asked about their gender and their occupation. The occupational background was diverse with 36 different occupations named by the participants.

### Measures

All English items were translated into German by two native speakers using the translation retranslation method. This means one native speaker translated the item into German and the other translated this item back into English. This way we were able to check if the translation was correct.

#### Workforce Agility I

We used the 10 dimensions of the workforce agility model by Petermann and Zacher (2021) to design a measure for workforce agility. For each dimension, 5 to 9 items were generated by the first author on the basis of the exact definition and the subcategories in the model. The items were then checked by the second author. An exception to this was the measurement of self-organization. We used the self-organization scale of Rousseau and Aubé (2010) as it showed a good fit and high reliabilities in previous studies. The self-organization scale was adapted in order to fit an individual scale. This procedure resulted in a measure of 68 questions for the concept of workforce agility. The participants of the first sample were asked to rate these questions in regard to their daily work using a 5-point scale from 1 (seldom) to 5 (very often /always). We then proceeded to calculate the item intercorrelations, the correlations of each item with the average of the other items belonging to the scale, and we conducted a confirmatory factor analysis with Sample 1. Subsequently, we selected the three items with the best fit, taking intercorrelations, correlations with the scale score, and factor loadings of every dimension into account, choosing the highest score while checking that the score did not exceed 0.9. All intercorrelations were in the range between 0.27 and 0.83 with a mean of 0.51 and a SD of 0.16. Furthermore, we compared the results of several confirmatory factor analyses to test whether the 10 dimensions proposed by Petermann and Zacher (2021) are distinct dimensions of workforce agility. For this, we used several theoretically deduced models with nine or eight factors as well as a one-factor model (Table 3). The comparison of the different models showed that the proposed item structure with 10 factors was the best fit for the data [*χ*^2^(360) = 546.291; RMSEA = 0.049; CFI =0.925; TLI = 0.909]. This was validated again *via* a second confirmatory factor analysis using the second sample of 533 employees that showed support for the 10 dimensional structure of workforce agility [*χ*^2^(360) = 559.133; RMSEA = 0.032; SRMR =0.039 RCFI =0.961; RTLI = 0.953].

**Table 3:** Results confirmatory factor analysis sample 1.

	Test statistic	Df	*P*	RMSEA	CFI	TLI
One-factorial model	1737.937	405	<0.001	0.123	0.452	0.411
Nine factorial model (iteration and test one factor)	687.803	369	<0.001	0.063	0.871	0.847
Nine factorial model (decision and user one factor)	646.733	369	<0.001	0.059	0.887	0.886
Eight factorial model (iteration and test/learning and transparency one factor)	738.234	377	<0.001	0.066	0.853	0.831
Nine factorial model (transparency and learning one factor)	596.699	369	<0.001	0.053	0.908	0.891
Nine factorial model (collaboration and self-organization one factor)	630.606	369	<0.001	0.057	0.894	0.875
Nine factorial model (learning and self-organization one factor)	581.709	369	<0.001	0.051	0.914	0.898
Ten factorial model	546.291	360	<0.001	0.049	0.925	0.909

We chose a model of workforce agility that was formative instead of reflective. Contrary to a reflective measure, in formative measurement, the direction of the relationship runs from the measure to the construct and not vice versa (Diamantopoulos et al., 2008). Hence, a change of any of the dimensions changes the overall construct (Diamantopoulos and Siguaw, 2006). As we assumed that the theoretical construct of workforce agility is defined by a combination of the 10 observable behaviors and a change in one of these measures would result in a change of the overall construct and not vice versa, we decided to use a formative construct. We, therefore, aggregated the 10 dimensions into one overall score of workforce agility to avoid committing a Type I error due to the large number of statistical tests (Diamantopoulos and Siguaw, 2006). To aggregate the dimensions, we followed the approach of Edwards (2001) and added all dimensions up to create one aggregated causal construct of workforce agility. This led to an overall construct consisting of the sum of 10 dimensions described in the model of Petermann and Zacher (2021) measured by 3 items each (please see Table 4 for the complete scale). As it is not appropriate to compute internal consistency estimates for constructs with formative measures, we did not compute Cronbach’s alpha values (Edwards, 2001). However, we computed Cronbach’s alpha values, standard deviations, and means for the 10 dimensions of workforce agility. These are depicted in Table 5.

**Table 4:** Dimensions and items of the workforce agility scale.

Dimensions	Items	Factor loadings sample (1/2)
Accept changes	At work I can quickly adapt to different situations. I am able to assume different roles in my work. If necessary, I find it easy to react to changes.	(0.654 / 0.711) (0.741 / 0.621) (0.713 / 0.697)
Decision making	I often delay important decisions longer. (Inverted) I am already making decisions that lead to solutions for the problems of the future. I like to take responsibility for topics at work.	(0.398 / 0.340) (0.568 / 0.547) (0.639 / 0.692)
Create Transparency	I actively share all information I have. When I have a question I often go directly to the relevant person. I ask colleagues outside my immediate environment for new information.	(0.624 / 0.457) (0.417 / 0.543) (0.423 / 0.465)
Collaboration	I regularly show my appreciation for others. I can respond well to the feelings and emotions of others. I enjoy working together with others.	(0.739 / 0.713) (0.676 / 0.657) (0.695 / 0.607)
Reflection	At work I think about how things could be done differently. I question how we could improve our cooperation. I am looking for new possibilities and tools to improve my procedures and processes.	(0.818 / 0.789) (0.748 / 0.798) (0.818 / 0.759)
User centricity	Customer feedback is one of the most important things to improve our product. The customer is an important part of our project. The customer perspective is actively included in our decision-making processes.	(0.750 / 0.705) (0.882 / 0.826) (0.767 / 0.771)
Iteration	We constantly question our product in order to improve it. We try to develop the product step by step, so that we can always assess whether we are still on the right track. In unserem Entwicklungsprozess wechseln sich kurzzyklisch Entwicklung und Evaluation ab.	(0.814 / 0.802) (0.689 / 0.622) (0.689 / 0.660)
Testing	We test every product before we make it public. Without testing a product, we do not let it go on the market. Product tests are an integral part of our development process.	(0.913 / 0.892) (0.891 / 0.805) (0.859 / 0.819)
Self-organization	I monitor the results of my work. I am looking for better ways to do my job. I introduce new methods to do my work.	(0.418 / 0.431) (0.727 / 0.802) (0.744 / 0.781)
Learning	I place great value on always learning new things. I am constantly expanding my skills. It is important for me to expand my knowledge.	(0.656 / 0.816) (0.821 / 0.655) (0.645 / 0.700)

**Table 5:** Means, standard deviations, and Cronbach’s alpha in sample 2.

	Mean	SD	Cronbach’s alpha
Accepting changes	4.22	0.52	0.71
Decision making	3.79	0.59	0.52
Creating Transparency	4.02	0.50	0.49
Collaboration	4.14	0.57	0.69
Reflection	4.05	0.65	0.82
User centricity	4.04	0.77	0.81
Iteration	3.43	0.73	0.74
Testing	3.88	1.01	0.88
Self-organization	4.05	0.61	0.70
Learning	4.34	0.54	0.76

#### Workforce Agility II

Furthermore, we used the workforce agility scale from Cai et al. (2018) as a second measure of workforce agility. This scale is the latest version of the most popular scale in workforce agility research, which was originally developed by Sherehiy (2008). According to a recent literature review, adaptions of Sherehiy’s scale have been used in 13 out of 31 studies examining agility (Salmen and Festing, 2021). The scale uses a 5-point Likert scale from 1 (strongly disagree) to 5 (strongly agree) and consists of 15 items divided in the three dimensions proactivity, adaptivity, and resilience. Examples of the items are “I am trying to find out more effective ways to perform my job,” “In my work, I can accept critical feedback,” and “I am able to perform my job efficiently in difficult or stressful situations.” Cronbach’s alpha for the dimension proactivity 0.61, for the dimension adaptivity 0.80, and for resilience 0.68.

#### Workforce Agility III

Additionally, we used the workforce agility scale from Braun et al. (2017). This scale has also been shown to have a good reliability and validity. It consists of 5 items and is measured on a 5-point Likert scale ranging from 1 (strongly disagree) to 5 (strongly agree). Examples for these items include as: “I am always thinking about what we need to do differently to meet upcoming change” or “In the last month, I have proposed a change about our work to my leader.” Cronbach’s alpha for this study is 0.86.

#### Job Performance Scale

We use the performance scale of Welbourne et al. (1998). This performance scale divides performance into different roles: (a) the task role, concerning the quality, quantity, customer service, and accuracy of work, (b) the organization role, concerning promoting the company, helping others, and work for the overall good of the company, and (c) the innovator role, coming up with and implementing new ideas, finding new ways of doing things, and creating better processes and routines. The scale provides a good fit for our study since it is based on theory, has been shown to be a reliable and valid measure, is relatively short, and has a good face value. Due to our hypotheses, we only used three of the dimensions of performance: (a) Task performance (4 items, e.g., “Accuracy of work”), (b) innovator performance (4 items, e.g., “Coming up with new ideas”), and (c) organizational citizenship behavior (4 items, e.g., “Working for the overall good of the company”). The response scale was a 5-point scale from 1 (needs much improvement) to 5 (excellent). Cronbach’s alpha in this study was 0.85 for task performance, 0.92 for innovator performance, and 0.90 for organizational citizenship behavior.

#### Job Satisfaction

Job satisfaction was evaluated using a one item scale by Wanous et al. (1997). It has been shown that a single item measure for job satisfaction is an acceptable alternative for a multiple item measure. The item we used was “How satisfied are you with your job as a whole?” and was measured on a 7-point scale ranging from 1 (very dissatisfied) to 7 (very satisfied).

#### Job Exhaustion

Job exhaustion was evaluated using three items from the Maslach burnout inventory (Maslach and Jackson, 1981). The items used were “I feel emotionally drained from my work,” I feel fatigued when I get up in the morning and have to face another day in the job,” and “I feel burned out from my work” and were measured on a 7-point scale ranging from 1 (never) to 7 (every day). Cronbach’s alpha in this study was 0.87.

### Analysis

We applied structural equation modeling with latent and manifest variables using the RStudio package lavaan to test our hypotheses. Structural equation modeling (SEM) was considered appropriate for our model since we wanted to test a complex model that contains several latent and mediating variables. SEM is a theory-driven method and an effective way to explain the relationship between multiple variables when the model that is tested is a path analytic model that consists of several latent constructs and mediating variables which are measured by multiple indicators (Tabachnick and Fidell, 2014). This analytic approach enabled us to examine the effects in relation to each other. Furthermore, it enabled us to show the complete model with all dependent and independent variables in one model. We used robust maximum likelihood (MLR) as estimator in our model, given that we saw some deviations from the normality criterion, and this estimator is relatively robust against nonnormality. The variables innovation performance, task performance, organizational citizenship behavior (four indicator variables each), and job exhaustion (three indicators) were added as latent variables to the model, job satisfaction was added as observed variable. We used the scale of Petermann and Zacher (2021) as independent variable for workforce agility and added it to the model as an observed variable that was constructed using the sum of all 10 dimensions of the workforce agility construct (Edwards, 2001). In a first step, we used confirmatory factor analysis to determine the fit of the aggregated scale. This was done by adding only the aggregated factor of agility and the outcome variables to the model and comparing this model to a model using all 10 workforce agility dimensions as independent variables. In a second step, we computed a full model adding the workforce agility construct of Braun et al. (2017) and the three-dimensional construct of Cai et al. (2018) as measure for workforce agility. For this, we added the dimensions of Braun et al. (2017; 5 indicators) and of Cai et al. (2018; proactivity 5 indicators, adaptability 6 indicators, and resilience 4 indicators) as latent variables to the model. Latent variables in all the models were allowed to correlate. The fit of our model was evaluated using the chi-square statistic, root square error of approximation (RMSEA) below 0.06 (Hu and Bentler, 1999), the comparative fit index (CFI) above 0.95, the standardized root mean squared residual (SRMR) below 0.08, and the Tucker-Lewis Index (TLI) above 0.95 (Bentler and Bonett, 1980).

## Results

The aggregated construct for workforce agility correlated highly with the dimensions of the workforce agility scale of Cai et al. (2018; proactivity *r* = 0.59, *p* < 0.001, adaptability *r* = 0.62, *p* < 0.001, and resilience *r* = 0.48, *p* < 0.001) and the workforce agility scale of Braun et al. (2017; *r* = 0.62, *p* < 0.001). Table 6 presents descriptive statistics and the correlation matrix for all variables used in the regression model.

**Table 6:** Means, standard deviations, correlations, and Cronbach’s alphas for Sample 2.

	*Mean*	*SD*	1	2	3	4	5	6	7	8	9	10
1. Workforce agility (based on Petermann and Zacher, 2021)	40.1	3.80	-									
2. Innovative performance	3.88	0.71	0.44**	(0.92)								
3. Task performance	4.15	0.56	0.30**	0.63**	(0.85)							
4. Organizational citizenship behavior	4.15	0.60	0.35**	0.67**	0.65**	(0.90)						
5. Job exhaustion	2.65	1.15	−0.15**	−0.18**	−0.11*	−0.21**	(0.87)					
6. Job satisfaction	5.55	1.16	0.16**	0.23**	0.16**	0.20**	−0.23**	-				
7. Workforce agility (Braun et al., 2017)	3.49	0.75	0.62**	0.42**	0.24**	0.27**	−0.13	0.12**	(0.86)			
8. Proactivity	4.21	0.45	0.59**	0.33**	0.25**	0.29**	−0.23**	0.11**	0.46**	(0.61)		
9. Adaptability	4.15	0.46	0.62**	0.38**	0.37**	0.36**	−0.19**	0.11**	0.43**	0.54**	(0.80)	
10. Resilience	3.93	0.51	0.48**	0.31**	0.26**	0.26**	−0.16**	0.06	0.35**	0.45**	0.58**	(0.68)

SD is used to represent standard deviation. Proactivity, adaptability, and resilience were measured with the workforce agility scale by Cai et al. (2018).

* *p* < 0.05;

** *p* < 0.01.

The model examines relation of workforce agility on five different outcome variables. We hypothesized that workforce agility is directly related to the job-related factors innovative performance, task performance, organizational citizenship behavior, job exhaustion, and job satisfaction. The first model using only the aggregated factor of workforce agility as independent variable indicated a good fit [*χ*^2^(106) = 270.469; RMSEA = 0.058; SRMR = 0.033; RCFI = 0.966; TLI = 0.957]. We then compared this model to a model in which we placed all 10 workforce agility dimensions as independent variables [*χ*^2^(885) = 1450.567; RMSEA = 0.036; SRMR = 0.043; CFI = 0.946; TLI = 0.937]. The comparison of the models using a chi-square difference test shows that the model with the aggregated indicator of workforce agility shows the better fit [*χ*^2^(779) = 1171.7, p < 0.001]. This is in line with our prediction that workforce agility is a multidimensional construct and we proceeded to use the aggregated workforce agility construct to calculate the full model. The full model using all three workforce agility measures did only show a marginal fit [*χ*^2^(590) = 15414.945; RMSEA = 0.055; SRMR = 0.101; RCFI = 0.895; RTLI = 0.881; Table 7]. This was expected in the design since we used three scales measuring the same construct. We will proceed to use the values from the full model to gain a direct comparison of the three measures in terms of their predictive validity. We will, however, shortly mention if each measure taken separately is significantly related to the outcome of every hypothesis. To get an exact depiction of the beta values and significance levels for each model, please see Tables A1–A3 in the Appendix.

**Table 7:** Model fits for the CFA with sample 1 and sample 2 and the regression analysis.

	Test Statistic	Df	*P*	RMSEA	CFI	TLI
CFA Sample 1 Workforce agility	546.291	360	< 0.001	0.050	0.925	0.909
CFA Sample 2 Workforce agility	559.133	360	< 0.001	0.032	0.961	0.953
CFA Sample 2 Regression Analysis	1541.945	590	< 0.001	0.058	0.895	0.881

Hypothesis 1 predicted that workforce agility is significantly related to the performance dimensions (a) task performance, (b) organizational citizenship behavior, and (c) innovative performance. All three workforce agility measures were significantly related to all three performance dimensions when taken separately. When added in the same model only the adaptability (*B* = 0.553, *SE* = 0.193, *β* = 0.355, *z* = 2.865, *p* = 0.004) and the resilience (*B* = 0.153, *SE* = 0.077, *β* = 0.154, *z* = 2.002, *p* = 0.045) dimension of the model of Cai et al. (2018) significantly related to task performance. The adaptability dimension of Cai et al. (2018; *B* = 0.485, *SE* = 0.195, *β* = 0.315, *z* = 2.488, *p* = 0.013) and the measure of Petermann and Zacher (2021; *B* = 0.027, *SE* = 0.011, *β* = 0.178, *z* = 2.564, *p* = 0.010) significantly related to organizational citizenship behavior, and only the workforce measure of Petermann and Zacher (2021; *B* = 0.030, *SE* = 0.011, *β* = 0.169, *z* = 2.719, *p* = 0.007) and of Braun et al. (2017; *B* = 0.302, *SE* = 0.079, *β* = 0.262, *z* = 3.797, *p* < 0.001) significantly related to innovative performance.

According to Hypothesis 2, workforce agility is positively related to (a) job satisfaction and negatively related to (b) job exhaustion. When taken separately, the measure of Petermann and Zacher (2021) and the measure of Braun et al. (2017) were positively related to job satisfaction and the measure of Petermann and Zacher (2021) and the resilience dimension of the measure of Cai et al. (2018) were significantly related to job exhaustion. When added in the same model, no measure significantly related to job satisfaction and only the resilience component of the model of Cai et al. (2018) significantly related to job exhaustion (*B* = −0.327, *SE* = 0.143, *β* = −0.178, *z* = −2.286, *p* = 0.022) (Table 8).

**Table 8:** Results of regression analyses.

Variables	*B*	*SE*	*β*	*z*	*p*	R^2^
**Innovative performance**						0.209
Proactivity	−0.004	0.165	−0.003	−0.027	0.978	
Adaptability	0.194	0.216	0.106	0.901	0.368	
Resilience	0.167	0.096	0.143	1.730	0.084	
Workforce agility (Braun et al., 2017)	0.302	0.079	0.262	3.797	< 0.001	
Workforce agility (Petermann and Zacher, 2021)	0.030	0.011	0.169	2.719	0.007	
**Task performance**						0.175
Proactivity	−0.224	0.139	−0.179	−1.606	0.108	
Adaptability	0.553	0.193	0.335	2.865	0.004	
Resilience	0.153	0.077	0.154	2.002	0.045	
Workforce agility (Braun et al., 2017)	0.099	0.067	0.101	1.480	0.139	
Workforce agility (Petermann and Zacher, 2021)	0.009	0.010	0.059	0.876	0.381	
**Organizational citizenship**						0.138
Proactivity	−0.236	0.146	−0.191	−1.621	0.105	
Adaptability	0.485	0.195	0.315	2.488	0.013	
Resilience	0.079	0.075	0.081	1.054	0.292	
Workforce agility (Braun et al., 2017)	0.120	0.069	0.124	1.738	0.082	
Workforce agility (Petermann and Zacher, 2021)	0.027	0.011	0.178	2.564	0.010	
**Exhaustion**						0.060
Proactivity	−0.079	0.245	−0.034	−0.322	0.747	
Adaptability	−0.292	0.315	−0.101	−0.930	0.352	
Resilience	−0.327	0.143	−0.178	−2.286	0.022	
Workforce agility (Braun et al., 2017)	0.223	0.143	0.122	1.560	0.119	
Workforce agility (Petermann and Zacher, 2021)	−0.016	0.021	−0.055	−0.734	0.463	
**Work satisfaction**						0.021
Proactivity	0.332	0.291	0.119	1.140	0.254	
Adaptability	−0.091	0.345	−0.026	−0.265	0.791	
Resilience	−0.006	0.165	−0.002	−0.034	0.973	
Workforce agility (Braun et al., 2017)	−0.030	0.176	−0.014	−0.172	0.864	
Workforce agility (Petermann and Zacher, 2021)	0.039	0.024	0.114	1.629	0.103	

Proactivity, adaptability, and resilience were measured with the workforce agility scale by Cai et al. (2018).

## Discussion

The aim of this study was to develop a new measurement of workforce agility based on the model of Petermann and Zacher (2021), compare this model to two established workforce agility measures of Braun et al. (2017) and Cai et al. (2018) in regard to their predictive validity and model fit, and to examine the hypothesis that workforce agility has positive relationship with work-related outcomes. The literature to date has suggested several factors that are related to workforce agility, but empirical testing has been sparse. Our findings support the previous literature about workforce agility in that they provide empirical evidence for the suggested relations of workforce agility with work-related outcomes. As hypothesized innovative performance, organizational citizenship behavior, task performance, job satisfaction, and job exhaustion were all found to be related to workforce agility.

The construction and validation of the measurement for workforce agility add to the previous literature in that it provides a specific measurement based on 10 different dimensions that shows a good model fit and high predictive validity toward work-related outcomes. An advantage of the new model is that it was constructed based on an open and integrating approach presenting an opportunity to integrate several models and conceptualizations. It, thus, answers the call of Sherehiy (2008) that asked for a model and measure with several specified dimensions instead of three more global ones. In contrast to other measures, this measurement is based on 10 specific behaviors which might offer new insights into the exact components of workforce agility that influence certain outcomes and could, therefore, allow for more detailed inferences about the relations of single dimensions with different outcome variables in the future. At the same time, the measure provides an aggregated factor that strongly correlates with previously validated measures of workforce agility and can, thus, be used to measure workforce agility in a more general term.

The comparison of the new measure with the two established workforce agility scales of Braun et al. (2017) and Cai et al. (2018) showed mixed results. All three measures correlated highly with each other and showed significant relations with the outcome variables when considered separately. When compared in the same model, however, different strength and weaknesses became apparent. Arguably, the most important outcome of agile workforce behavior innovative performance was, for example, only significantly related to the scale of Petermann and Zacher (2021) and the scale of Braun et al. (2017), whereas task performance and job exhaustion only related to the model of Cai et al. (2018). In general, the model of Cai et al. (2018) seemed to be the best predictor for most of the chosen outcome variables with the exception of innovative performance. All the scales showed a good model fit in the separate models. We want to note, however, that the confirmatory factor analysis of the model of Cai et al. (2018) did not show a good fit. This fit could be improved by deleting the two inverted items of the proactivity dimensions as well as item four of the resilience dimension. Furthermore, it should be noted that the length of the scales differed significantly.

Additionally, this research now provides insights into the relation of three different performance factors with workforce agility. Particularly, a positive relation with innovative performance has often been hypothesized due to an agile workforces ability to cope well with new situations, master uncertainty, learn and acquire new skills quickly, and find good solutions for new problems (Braun et al., 2017; Doeze Jager-van Vliet, 2017; Harsch and Festing, 2020). The results of our study support this hypothesis and show that there is a strong positive relation between workforce agility and innovative performance. We also found a positive relation of workforce agility with task performance and organizational citizenship behavior. This is also in line with previous research that suggested that an agile workforce produces higher quality of work (Mann and Maurer, 2005), is better able to observe, anticipate, and meet the need of a customer (Chonko and Jones, 2005), and collaborates better across functional, team, and department boarders to utilize all existing resources (Sherehiy and Karwowski, 2014).

Other positive work outcomes that were found to be related to workforce agility included job exhaustion and work satisfaction. Job exhaustion was found to be negatively, and job satisfaction positively related to workforce agility. These relations, however, were much weaker than the relations of workforce agility with the performance dimension and, in the case of job satisfaction, vanished when all three workforce agility measures were added in the same model. Our findings are in line with the hypothesis that workforce agility positively relates to well-being (Mannaro et al., 2004; Tuomivaara et al., 2017). However, as the effects are small and the literature to date is somewhat ambiguous more research is needed to gain a better understanding.

### Theoretical Implications

The results of this study may have implications for further theory development in three ways. First, the newly proposed measure integrates a more general approach into a very specific measurement and can be applied in diverse settings and for different research questions. Taken together with a good model fit and a good predictive validity, we suggest that this scale could be good alternative for measuring workforce agility. Second, we argue that this research adds to the literature in that it tests and expands the agility theories of Sherehiy (2008) and Braun et al. (2017). Even though agility theories have often been criticized for their model fit as well as their conceptual vagueness, the measures showed a good fit and predicted the proposed outcome variables well. They often criticized three-dimensional model, tested with the measure of Cai et al. (2018), and did show some problems with model fit; however, these problems could be resolved by deleting three items from the scale. Adapting this measure might be a valuable next step in the agility literature since these adaptions seem to fix issues with model fit that did arise in previous studies. This might be especially beneficial since it appeared to be the best predictor of the outcome variables with the exception of innovative performance.

Third, we argue that the concept of workforce agility should be included in future conceptualizations of criteria of performance and especially innovative performance at work. As predicted workforce agility was related to the three considered performance dimensions of the performance model of Welbourne et al. (1998), task performance, innovative performance, and organizational citizenship behavior. We suggest future research could advance the understanding of the relation between agility and performance further by considering performance and especially innovative performance as an outcome of workforce agility. At the same time, our research adds to the innovation literature in that it provides evidence for the proposed relation between workforce agility and innovative performance. Research on team innovation has identified the two main factors that promote a climate for innovation: support for innovation and a climate for excellence (West et al., 2003; Eisenbeiss et al., 2008). We argue that an agile workforce actively creates a climate of innovation in which employees feel free to take initiative, explore, and develop new ideas, as well as regularly test and adapt their products. Thus, workforce agility could be seen as an underlying factor supporting a climate of innovation.

### Practical Implications

Our research has might have valuable consequences for organizational practice in two main ways. Organizations can use the measurement to specifically access their current level of agility and organizations can adapt their training and development processes to increase workforce agility to foster positive work outcomes such as a better performance or a higher job satisfaction.

The workforce agility scale constructed and validated by this research could be used in the organizational context in several ways. It could be used in organizational development to assess the level of workforce agility in the organization or in single departments. An assessment based on the specific dimensions of the validated measurement will give an organization an exact depiction of their current workforce agility level in each dimension. This way organizations or departments could tailor development processes exactly to their current agility level and increase the coherence with their agility needs. Modular training could be developed to strengthen the skills of employees in single workforce agility dimensions and increase their overall agility. Moreover, structures and processes could be introduced in order to strengthen single dimensions of workforce agility. An example for this could be to integrate a personalized decision process to improve and speed up decisions. Furthermore, the workforce agility measure might be used by companies during the recruitment process to assess the level of workforce agility for job applicants. This way organizations will be able to select candidates that show a high level of workforce agility and, therefore, show a good fit to or even increase the agility level of the selected position. This could be especially effective if the agility need of the position is assessed before the recruitment process and the agility level of the applicant can be compared to this need.

### Limitations and Future Research

This study has a number of limitations which mandate a certain degree of caution when interpreting the results. The first limitation is the cross-sectional design of the study that does not allow for an investigation of the relationship between workforce agility and the outcome variables over time. To tackle this limitation, for at least the validation of the measurement, we compared the results of the confirmatory factor analysis between the two samples. Nevertheless, future research should use longitudinal or experimental study design to investigate the relations between workforce agility and performance, job satisfaction, and job exhaustion. Another limitation concerns the items of the newly created measure. The items measuring the concepts “decision making” and “creating transparency” had low Cronbach’s alpha values. It might be fruitful for future research to revise these items to strengthen internal consistency. Lastly, the study is limited by the composition of the sample. The greatest part of the participants came from one manufacturing company in Germany. It might therefore be interesting to validate our findings with a more diverse sample and in other countries.

As the workforce agility literature is still in its early stages, there are several aspects that should be considered by future research. First, contrary to previous conceptualizations, this research defines agility as formative instead of reflective construct. This means that we expect the causality to run from the dimensions toward the agility construct and not vice versa. Together with the fact that we define agility as a multidimensional behavioral taxonomy consisting of 10 behaviors, this approach could initiate a discussion about the causal effects of workforce agility. Future research should further examine the nature and causal effects of the agility construct.

Second, we expect self-determination theory to mediate the relationship between workforce agility and performance as well as its relationship to well-being. Future research examining this question could provide a better understanding of the causality of the effects that were found by this research. It would also be interesting to use an experimental design to look at different agility interventions and their influence on performance and well-being over time. Different interventions could be used to examine their influence on agility and, consequently, the output factors.

Third, future research could further explore the relation between workforce agility and innovation. We suggest that an agile workforce actively creates a climate of innovation which in turn leads to higher innovative performance. Examining this question could lead to a better understanding of the relation between agility and innovative performance and add to the current innovation literature.

Fourth, future research could also look at targeted training and interventions to examine the individual influence of the interventions on agility dimensions as well as on the outcome factors. This could be paired with an experimental study design in which different dimensions are manipulated to compare their impact on agility and organizational functioning. We believe that this study provides a good basis for further investigations in the field of workforce agility. It contributes to the literature by comparing different models and measures of workforce agility and by linking it to performance and well-being.

## Conclusion

In conclusion, these findings expand previous literature in that they provide empirically measured evidence of the relationship between workforce agility and positive work outcomes. Especially the positive relationship between workforce agility and innovative performance was previously often suggested and could be confirmed by this research. This finding might be particularly important for practitioners who are currently restructuring their organization to become more agile. It may also have implications for the scientific practice as it shows that agility could be included into future conceptualizations of innovative performance. Additionally, this study developed a new multidimensional measure of workforce agility. In a second step, this research compared the new model to different workforce agility measures on the basis of their fit and their predictive validity and discussed how to develop the scales further. We argue that this research could be a good basis for future research in the field of workforce agility as it offers a clear comparison of different agility measures and provides empirical evidence of the relation between workforce agility and positive work outcomes.

## Data Availability Statement

The data is only available on request and under individual consideration.

## Ethics Statement

The studies involving human participants were reviewed and approved by the responsible board at the Daimler AG (Betriebsrat Untertürkheim). The patients/participants provided their written informed consent to participate in this study.

## Author Contributions

MP and HZ contributed to the conception and design of the research. MP conducted the research and statistical analysis, wrote the first draft of the manuscript, and revised the manuscript following feedback. HZ provided feedback and revised the first draft and subsequent versions of the manuscript. All authors contributed to the article and approved the submitted version.

## Funding

This research was supported by Open Access Publishing Fund of Universität Leipzig and German Research Foundation (DFG).

## Conflict of Interest

MP was employed by the company Daimler AG.

The remaining authors declare that the research was conducted in the absence of any commercial or financial relationships that could be construed as a potential conflict of interest.

## Publisher’s Note

All claims expressed in this article are solely those of the authors and do not necessarily represent those of their affiliated organizations, or those of the publisher, the editors and the reviewers. Any product that may be evaluated in this article, or claim that may be made by its manufacturer, is not guaranteed or endorsed by the publisher.

## Appendix

**A1:** Results of the regression analyses using only the scale of Petermann and Zacher (2021).

Variables	*B*	*SE*	*β*	*z*	*p*	R^2^
**Innovative performance**						0.202
Workforce agility (Petermann and Zacher, 2021)	0.085	0.009	0.450	9.197	< 0.001	
**Task performance**						0.094
Workforce agility (Petermann and Zacher, 2021)	0.047	0.008	0.306	5.754	< 0.001	
**Organizational citizenship**						0.131
Workforce agility (Petermann and Zacher, 2021)	0.056	0.008	0.361	6.952	< 0.001	
**Exhaustion**						0.024
Workforce agility (Petermann and Zacher, 2021)	−0.044	0.013	−0.154	−3.284	0.001	
**Work satisfaction**						0.027
Workforce agility (Petermann and Zacher, 2021)	0.056	0.015	0.163	3.691	< 0.001	

**A2:** Results of the regression analyses using only the scale of Braun et al. (2017).

Variables	*B*	*SE*	*β*	*z*	*p*	R^2^
**Innovative performance**						0.221
Workforce agility (Braun et al., 2017)	0.574	0.068	0.479	8.498	< 0.001	
**Task performance**						0.073
Workforce agility (Braun et al., 2017)	0.270	0.057	0.271	4.731	< 0.001	
**Organizational citizenship**						0.096
Workforce agility (Braun et al., 2017)	0.312	0.057	0.310	5.428	<0.001	
**Exhaustion**						0.005
Workforce agility (Braun et al., 2017)	−0.124	0.093	−0.068	−1.338	0.181	
**Work satisfaction**						0.016
Workforce agility (Braun et al., 2017)	0.285	0.111	0.128	2.561	0.010	

**A3:** Results of the regression analyses using only the scale of Cai et al. (2018).

Variables	*B*	*SE*	*β*	*z*	*p*	R^2^
**Innovative performance**						0.227
Proactivity	0.306	0.144	0.198	2.125	0.034	
Adaptability	0.337	0.220	0.175	1.531	0.126	
Resilience	0.209	0.100	0.172	2.100	0.036	
**Task performance**						0.196
Proactivity	−0.135	0.120	−0.106	−1.129	0.259	
Adaptability	0.602	0.192	0.379	3.128	0.002	
Resilience	0.171	0.076	0.170	1.332	0.025	
**Organizational citizenship**						0.172
Proactivity	−0.082	0.122	−0.064	−0.674	0.500	
Adaptability	0.611	0.197	0.383	3.107	0.002	
Resilience	0.101	0.076	0.101	1.332	0.183	
**Exhaustion**						0.067
Proactivity	0.098	0.202	0.042	0.486	0.627	
Adaptability	−0.371	0.301	−0.127	−1.233	0.217	
Resilience	−0.333	0.140	0.181	−2.382	0.017	
**Work satisfaction**						0.027
Proactivity	0.413	0.225	0.146	1.838	0.066	
Adaptability	0.054	0.338	0.015	0.160	0.873	
Resilience	0.029	0.164	0.013	0.174	0.862	

Proactivity, adaptability, and resilience were measured with the workforce agility scale by Cai et al. (2018).
